# Structure of Epigeic and Arboreal Ant Communities in Forest Fragments Within Agricultural Landscapes of the Brazilian Cerrado

**DOI:** 10.3390/insects17060656

**Published:** 2026-06-22

**Authors:** Helivelton H. M. Araújo, Vagner S. Vale, Flávio G. Jesus, Ednaldo C. Rocha, Carlos de M. Silva-Neto, André C. S. Almeida, Matheus C. Heinzelmann, Márcio S. Araújo

**Affiliations:** 1 Postgraduate Program in Plant Production, State University of Goiás, Ipameri 75780-000, GO, Brazil; heliveltonlive@outlook.com (H.H.M.A.); vagner.vale@ueg.br (V.S.V.); flavio.jesus@ifgoiano.edu.br (F.G.J.); ednaldo.rocha@ueg.br (E.C.R.); carlos.melo@ueg.br (C.d.M.S.-N.); matheuscarneiroh@gmail.com (M.C.H.); 2 Goiano Federal Institute, Urutaí Campus, Urutaí 75790-000, GO, Brazil; andre.almeida@ifgoiano.edu.br; 3 Department of Entomology and Acarology, University of São Paulo, Piracicaba 13418-900, SP, Brazil

**Keywords:** Formicidae, guilds, richness, stratification, similarity, bioindicators

## Abstract

A large portion of the Brazilian Cerrado vegetation currently exists as small, disturbed, and isolated fragments. Evaluating the degree of anthropogenic disturbance in certain environments has been successfully achieved using bioindicator insects, particularly ants. This study aims to characterize the epigeic and arboreal ant communities in Cerrado fragments in the southeastern region of Goiás, bordered by agricultural crops, during both the dry and wet seasons, and to correlate these ant communities with the structural characteristics of the forest fragments. Morphospecies richness and ant community structure across the different environments were compared, and bioindicator species were proposed using the Indicator Value (*IndVal*) method. The ant community structure under the evaluated conditions was significantly altered, indicating effects that extend beyond fragment-specific variables and are associated with the surrounding agricultural landscape. The *IndVal* analysis suggested two environmental quality indicator species for the ground stratum: *Pheidole* cf. *radoszkowskii* and *Crematogaster stollii*. However, it did not point to any significant indicator species for the arboreal stratum. Ants mediate various functional roles and shifts in the structure of local communities, reinforcing the importance of these insects as bioindicators of environmental quality.

## Simple Summary

The reduction in biodiversity is directly linked to ecosystem loss and fragmentation, which have modified the Brazilian Cerrado landscape, transforming natural ecosystems into agroecosystems. Ants serve as biological indicators of environmental quality, offering a rapid and low-cost assessment tool. Using baited traps, we characterized the epigeic and arboreal ant communities within the Brazilian Cerrado agricultural landscape, relating their richness and composition to the structural characteristics of the forest fragments. The structure of the ant communities under the evaluated conditions was significantly altered, indicating effects that extend beyond fragment-level variables and are driven by the surrounding agricultural landscape. Importantly, ants mediate various functional roles; therefore, changes in local community structure imply alterations in broader ecosystem processes, underscoring the value of these insects as key bioindicators.

## 1. Introduction

The Brazilian Cerrado is currently highly fragmented, forming landscapes that include agroecosystems exerting strong agricultural pressure on natural remnants [1,2]. Thus, it becomes relevant to understand how forests, or vegetation remnants, are constituted and their role in the ecosystem. Understanding their structure allows for the development of action plans for their conservation, the conservation of genetic resources and similar areas, and the recovery of degraded and/or fragmented areas [3,4]. Vegetation fragmentation introduces a series of new factors into the evolutionary history of natural plant and animal populations. According to Viana and Pinheiro [5], differentially affect the demographic parameters of mortality and natality across species and, consequently, the structure and dynamics of ecosystems.

Fragmented landscapes exhibit consistent reductions in both local and regional diversity compared to continuous areas. Although the variance among fragments might seem higher in subdivided terrains, this increase is purely a consequence of the geographical distance between the remnants and does not compensate for the overall loss of species [6]. These authors challenge the idea that multiple small fragments can sustain the same biological richness as large, intact forests, emphasizing that maintaining continuous habitats and connectivity is fundamental to halting biodiversity decline.

The degree of isolation of the fragments is a crucial variable to consider, as it is known to promote a reduced flow of individuals; however, this is not the sole factor separating or connecting spatially divided populations. The spatial arrangement of forested areas within the environment is also of fundamental importance for the presence or absence of certain populations, especially when the original habitat is reduced to low proportions [7].

The Brazilian Cerrado possesses high plant species richness and high spatial heterogeneity, characteristics that contribute to its classification as the world’s most biodiverse tropical savanna [8]. The clearance and fragmentation of this biome over slightly more than five decades of large-scale anthropogenic occupation (primarily for pasture formation and agriculture) have resulted in a process of plant and animal biodiversity loss that threatens its sustainability and limits its future economic and social opportunities [7].

The environment of our study is the “dry forest” (*floresta seca* or *mata seca*) of the Cerrado in the southeastern region of Goiás, Brazil. Although the number of phytophysiognomies in the Brazilian Cerrado biome as a whole varies, three major groups of formations are recognized: forest (*cerradão* and dry forest), savanna (*cerrado sensu stricto* and *cerrado ralo*), and grassland (*campo sujo*, *campo cerrado*, and *campo limpo*) [9]. In this region, landscapes mostly consist of mosaics of agroecosystems and native vegetation areas in various shapes, sizes, and stages of conservation.

Habitat fragmentation also promotes significant abiotic changes in forest ecosystems. For instance, Lima-Ribeiro [10] reported a significant temperature variation between the edge and the interior of forest fragments. Furthermore, fragments are more susceptible to turbulence caused by winds, which, combined with high temperatures, accelerates water loss rates through evaporation [11] and reduces relative humidity [12]. Habitat reduction and fragmentation have altered diversity at local, regional, and global scales [13], while increased isolation among populations, alongside environmental changes, affects many ecological processes of populations and communities [14].

Forest biodiversity conservation depends on the interactions occurring between fragmented native forest areas and the surrounding matrix, which will determine the long-term persistence of species in these environments [15]. The effect of the matrix surrounding fragmented native areas on biodiversity is evident and well-documented in the literature [14,16,17]. Depending on its structure and composition, this matrix can offer food resources. Some animal species are capable of exploiting a matrix, or at least tolerating its effects, and their populations may remain stable or even increase in abundance during a fragmentation process [18]. Species that are more vulnerable to habitat fragmentation are those that do not tolerate changes in their habitat structure and rarely use the matrix. This topic is widely explored in birds [19]. For example, forest-dwelling birds unable to exploit the matrix are susceptible to population decline or even extinction [20].

According to Holanda et al. [21], understanding the processes resulting from habitat fragmentation, such as edge creation, is essential for developing strategies to recover forest fragments, which provides a foundation for reserve design and preventive measures to avoid species extinction. According to these authors, edge formation exposes species adapted to specific abiotic factors—previously found in the forest interior—to adverse factors, such as high temperatures, increased luminosity, higher wind speeds, and low relative humidity. Consequently, there is an increase in evapotranspiration, which influences the falling of larger trees. The presence of higher air and soil temperatures, and consequently lower relative humidity at the edges compared to fragment interiors, also highlights the influence of these edge effects [10]. The same author reported that the interiors of larger fragments are more similar to each other regarding vegetation structure, whereas the edges of smaller fragments are the ones that are most alike. The decrease in plant density towards larger fragments supports this hypothesis, indicating that the smaller the fragment, the greater the influence of microclimatic parameters in the interior due to the closer proximity to the edge, favoring the establishment and colonization of pioneer plant species.

Identifying rapid diagnostic tools to detect the degree of anthropization in this phytophysiognomy should be a constant pursuit, especially for proposing conservation measures. Bioindicators can assist in the rapid and mostly accurate diagnosis of environmental conditions. Research utilizing these environmental bioindicators has yielded positive results [14,22].

Insects are effective bioindicators of terrestrial anthropogenic disturbance [21]. Several studies have demonstrated that, among a variety of insect taxa, ants can potentially be used as environmental quality bioindicators [14,23,24,25,26,27]. Studies have already shown that ant communities can be different in the same region, depending on whether the vegetation cover is agricultural or natural forest formation [14]. They can even be influenced by the chemical characteristics of the soil, preferring more preserved and protected soils, with only groups already adapted (*Pheidole*, *Solenopsis* and *Acromyrmex*) to degraded environments remaining in exposed soils [23]. This is because ants present high abundance and species richness, include specialized taxa, have a broad geographic distribution, are easily sampled and separated into morphospecies with relative ease, and are sensitive to changes in environmental conditions [28].

Therefore, highlighting the relevance of agroecosystem dynamics and their interaction with the insect community, this study aimed to characterize the epigeic and arboreal ant communities in Cerrado fragments in the southeastern region of Goiás, bordered by agricultural crops, during the dry and wet seasons, to relate these ant communities to the structural characteristics of the forest fragments, and to suggest potential bioindicator species for these environments.

## 2. Materials and Methods

### 2.1. Study Area

**Figure 1:**
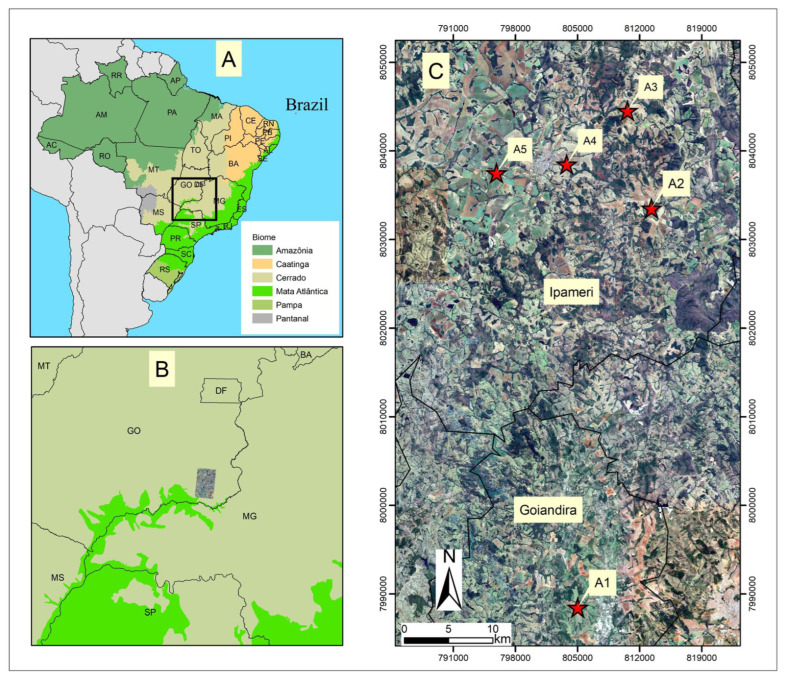
Geographic location of the study area at different scales (**A**–**C**). At a larger scale (**C**), the geographic coordinates of the investigated dry forest fragments are shown. Fragment A1, with an area of 8.6 ha, is located in the municipality of Goiandira, GO, while fragments A2, A3, A4, and A5, with areas of 53.7, 258.5, 42.5, and 21.3 ha, respectively, are located in the municipality of Ipameri, GO.

In this study, we selected and investigated five typical Cerrado vegetation fragments (dry forest, or *mata seca*), which are part of the legal reserves of private rural properties located in the rural areas of the municipalities of Ipameri and Goiandira, Goiás State, Brazil (Figure 1). In all selected fragments, the surrounding matrices were simultaneously occupied by the same crops (soybean in the main season and corn in the second season). To ensure relative sample independence, fragments were chosen with a minimum distance of 8.0 km between them (see fragments 4 and 5; Figure 1).

The investigated Cerrado fragments are located in the southeastern region of Goiás, Brazil. According to Alvares et al. [29], the climate of this region is characterized as a tropical savanna (Aw, according to the Köppen climate classification), with an annual precipitation ranging from 1600 to 1900 mm and an average annual temperature of approximately 23 °C. There are two well-defined seasons: a dry season from April to September, and a wet season from October to March.

Crop planting in the surrounding matrices of all Cerrado fragments was carried out using a no-tillage system, with sequential cropping. The first crop was planted between the second half of October and the first half of November, with the harvest occurring between the second half of February and the second half of March. The second crop, corn, was shown between the last week of February and the first half of April. In Brazil, these sequential cropping systems are referred to as *safra* (main crop) and *safrinha* (second or off-season crop). In our study, the corn harvest was conducted after the last ant sampling.

The assessment of land use and land cover (LULC) classes consisted of delimiting 1 km radius buffers, using the sampled points within the vegetation fragments as centroids, as proposed for other social insect groups [30]. These polygons were used as the spatial frame to extract the land use and land cover classes provided by Collection 9 of the MapBiomas Project [31]. Processing was performed in the R software (version 3.0.2) environment [32], utilizing the *terra* [33] and *sf* [34] packages. Raster values were extracted for each buffer and converted into area, considering the resolution of each pixel (30 m × 30 m = 900 m^2^).

### 2.2. Sampling of the Arboreal Component

To relate the structural characteristics of the arboreal plant community with the local epigeic and arboreal ant communities, a survey of the arboreal plants within the forest fragments was conducted. The list of arboreal species from the fragments located in the municipalities of Goiandira and Ipameri is presented in Table S1 (see Supplementary Materials).

In each fragment, three transects were randomly demarcated, containing ten 10 × 10 m plots, allocated from the edge towards the forest interior. Thus, a total of 30 plots of 10 × 10 m were evaluated. Forest species were identified using the Angiosperm Phylogeny Group IV (APG IV) [35] taxonomic system. Within these plots, the number of arboreal species and their basal area were quantified.

### 2.3. Ant Sampling

At the center of the previously described plots along the transect (from the edge to the fragment interior), spaced 10 m apart, one ground trap was placed, along with another trap attached to the nearest arboreal plant. This resulted in 30 traps per fragment in each evaluated season. The species composition of each point is the result of sampling with 3 traps per area and at each station. 3 × 5 × 2 = 30 traps per point. This effort was used both on the ground and in the tree.

The ground trap consisted of a 200 mL plastic cup, secured horizontally on the soil surface with a metal staple (to prevent it from being dragged by the wind), containing a food bait made of sardine oil, honey, and biscuit crumbs at the bottom. To capture ants directly from the trees, a trap with the same bait was attached to the tree trunk at a height of 1.30 m from the ground surface. These traps operated for a period of two consecutive hours during the morning (between 8:00 h and 10:00 h). After this period, the traps were retrieved from the field and taken to the entomology laboratory, where they were sorted and identified. This collection and sorting methodology followed the protocols adopted by Bernardes Júnior et al. [14].

It is important to note that our study considered only the capture frequency of morphospecies in each trap, calculated using the formula (a × 100)/*n*, where (a) represents the number of traps in which a certain species was captured and (*n*) represents the total number of traps (150). According to Benson & Harada [36], using capture frequency data avoids the overestimation of species that possess a high mass-recruitment capacity. To complement the data analysis, capture frequency data were used for descriptive community characterization and diversity estimates. In another approach, occurrence matrices were constructed separately for the *IndVal* analysis.

Ant identification was performed using the taxonomic keys proposed by Fernández [37] and Baccaro et al. [38], and through comparison with specimens from the reference collection at the Entomology Laboratory of UEG, UnU Ipameri. The classification of the ant community into functional groups was based on the works of Andersen et al. [39], Delabie et al. [40], Silvestre et al. [41], and Bernardes Júnior et al. [14].

Ant sampling during the wet season was carried out in early February (the region’s rainy period), when the surrounding matrix of the fragment was cultivated with soybean. Ant sampling during the dry season was conducted while the fragment matrices were still occupied by the *safrinha* corn, at the R5 and R6 phenological stages (dough and hard-dough stages), in early July (the region’s dry period).

### 2.4. Statistical Analysis

Ant richness (morphospecies and guild) across the different fragments, strata (ground and arboreal), and capture periods (wet and dry seasons) was estimated using the Jackknife 1 estimator to construct species accumulation curves (via interpolation and extrapolation). This approach represents species richness as a function of the sampling effort applied in each sampled buffer [42]. Using rarefaction and extrapolation methods via the iNEXT (iNterpolation/EXTrapolation) package [43], we evaluated the biodiversity and overall structure of the ant community in the forest fragments.

In this study, the use of the Jackknife 1 estimator in conjunction with the interpolation and extrapolation approach of the iNEXT (version 3.0.2) package presented fundamental advantages for the analysis of the richness and structure of the ant Community [44]. Jackknife 1 was employed because it is a non-parametric estimator based on the frequency of rare species (those that occur in only a single sampling unit), allowing for the correction of the underestimation of total richness resulting from finite sampling efforts, especially relevant in forest fragments with high environmental heterogeneity [45]. In parallel, iNEXT enabled standardization of comparisons of diversity across strata (soil and tree), fragments, and seasonal periods (dry and rainy) using rarefaction curves (interpolation) and extrapolation, eliminating bias from differences in sampling effort between locations.

The visualization of community structure and ant species composition across the investigated forest fragments was performed using Principal Coordinates Analysis (PCoA). To evaluate the effect of land use on the ant community, a Chi-square test was conducted, followed by a Correspondence Analysis (CA) linking land use attributes with ecological aspects of the ants. For ANOSIM and PERMANOVA, the occurrence/incidence of species in each transect, in each season (dry and rainy), and in each area was used. The Jaccard dissimilarity index was used as a measure of distance between samples. A vegan package was used for both analyses [45]. We used the Jaccard dissimilarity index with 999 permutations, where the areas (locations) are the groups compared by the analysis.

The relationship between the structuring variables of the Cerrado forest fragments (arboreal species richness and density, total basal area of the arboreal plot component, and trap distance from the edge) and ant species richness was analyzed using Principal Component Analysis (PCA). The correlation between the arboreal component and the ant community was performed only for the fragments in Ipameri, GO (A3, A4, and A5). The determination of habitat bioindicator ant species was conducted using the Indicator Value (*IndVal*) method proposed by Dufrêne and Legendre [44]. The predefined groups are soil and tree. The analysis was conducted to identify indicator species from these two environments. For *Indval*, the occurrence/incidence of species was used in each transect, in each season (dry and rainy), in each stratum (soil and tree), and in each area. For a second analysis approach using the *IndVal* test, reorganizing the samples containing the species records in each transect, in each season (dry and rainy), and in each area, grouping based on the results of the ordination by Correspondence Analysis (CA). Considering four landscape variables surrounding the studied fragments: percentage of area occupied by soybeans, pasture, forest, and savanna. After this new categorization, the *IndVal* index was used to identify the indicator species of each of these sample groups.

For each defined landscape use, the groups were, SOYBEANS: (1) samples from areas with more than 50% soybeans (A4 and A5) in the surroundings and (2) samples from areas with less than 50% soybeans (A1, A2 and A3); PASTURE: (1) samples from the area with more than 40% pasture (A1) and (2) samples from areas with less than 40% pasture (A2, A3, A4 and A5); FOREST: (1) samples from the area with more than 29% forest (A2) and (2) samples from areas with less than 29% forest (A1, A3, A4 and A5); and SAVANNA: (1) samples from areas with more than 8% savanna (A2 and A4) and (2) samples from areas with less than 8% savanna (A1, A3 and A5).

The morphospecies identified by *IndVal* as significant were identified to the species level to effectively serve as environmental indicator species. For species identification, the work of Rodrigues et al. [25] was used as a reference, and the specimens were stored in the entomological collection of the Entomology Laboratory of the State University of Goiás, Ipameri Unit.

All statistical analyses were performed in the RStudio software (2025.09.2) environment [32] utilizing the *vegan* [45], *iNEXT* [42,43], *indicspecies* [46], and *FactoMineR* [47] packages. Additionally, the *ggplot2* [48] and *factoextra* [49] packages were used for the graphical representation of the results.

## 3. Results

A total of 53 ant morphospecies were identified across the five Cerrado fragments, distributed among 6 subfamilies and 19 genera. The subfamily with the highest number of species was Myrmicinae, with 24 morphospecies, followed by Formicinae, with 14. The remaining subfamilies had three or four morphospecies. The most frequently captured genus was Pheidole, with 12 morphospecies. Along with Pheidole, the most commonly found genus in our study was *Camponotus* (Formicinae), with 11 morphospecies (Table 1).

**Figure 2:**
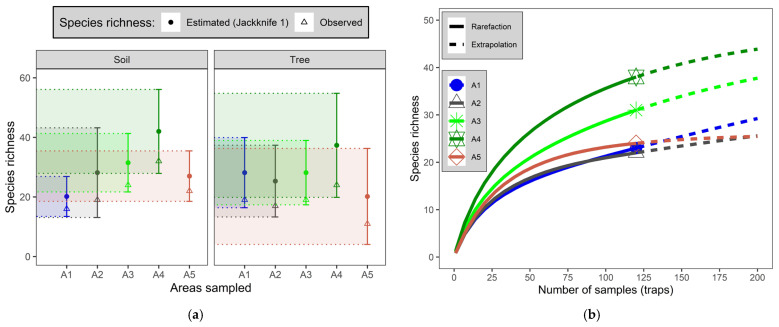
(**a**) Morphospecies richness of ants collected in trees and on the ground in each of the investigated forest fragments; (A1) in Goiandira and (A2, A3, A4, and A5) in Ipameri. The estimator used was Jackknife 1; the bars in the figure represent the 95% confidence interval adopted by the estimator (shaded areas). (**b**) Total estimated ant species richness with rarefied and extrapolated samples for 200 sampling units.

The observed and estimated ant richness in the ground and arboreal strata for individual fragments is presented in Figure 2a. The large range of variation shown by the confidence interval contributed to the lack of significant difference among most fragments in both studied strata. Rarefaction and extrapolation curves considering total species for isolated fragments highlight a higher estimated ant richness for fragments A4 and A3 (Figure 2b).

**Figure 3:**
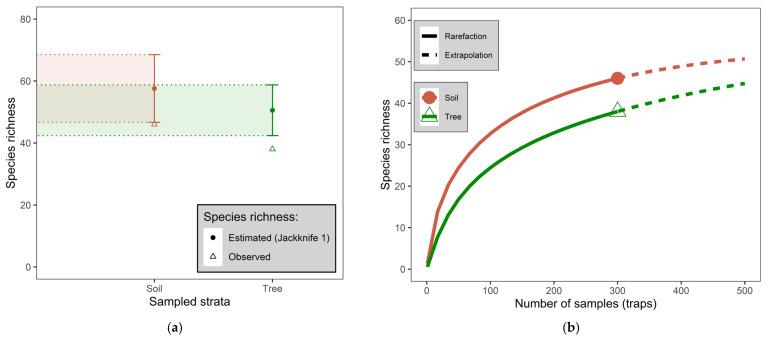
(**a**) Total morphospecies richness of ants collected on the ground and in trees across the five dry forest fragments. The estimator used was Jackknife 1; the bars in the figure represent the 95% confidence interval adopted by the estimator (shaded areas). (**b**) Total estimated ant species richness with rarefied and extrapolated samples for 500 sampling units.

**Figure 4:**
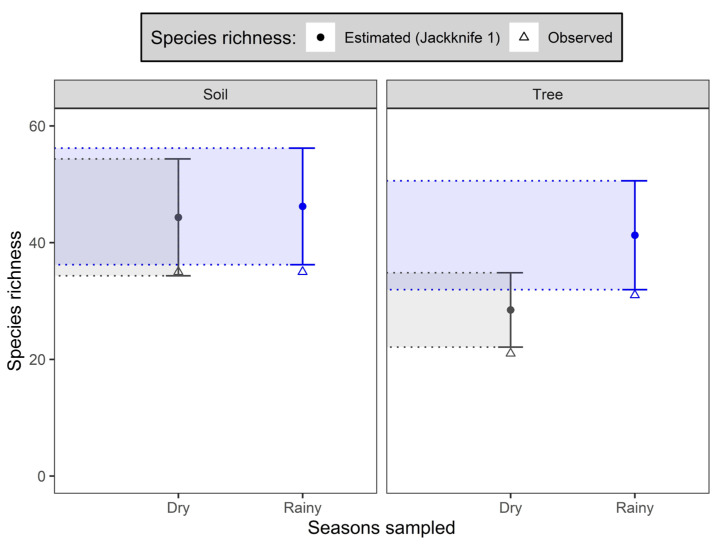
Morphospecies richness of ants captured in trees and on the ground in the five Cerrado fragments in the southeastern region of Goiás, during the dry and wet seasons. The estimator used was Jackknife 1. The bars in the figure represent the confidence interval adopted by the estimator, at *p* = 0.014 to tree, and *p* = 0.003 to soil (Vertical bars indicate the 95% confidence intervals for the estimated richness).

**Figure 5:**
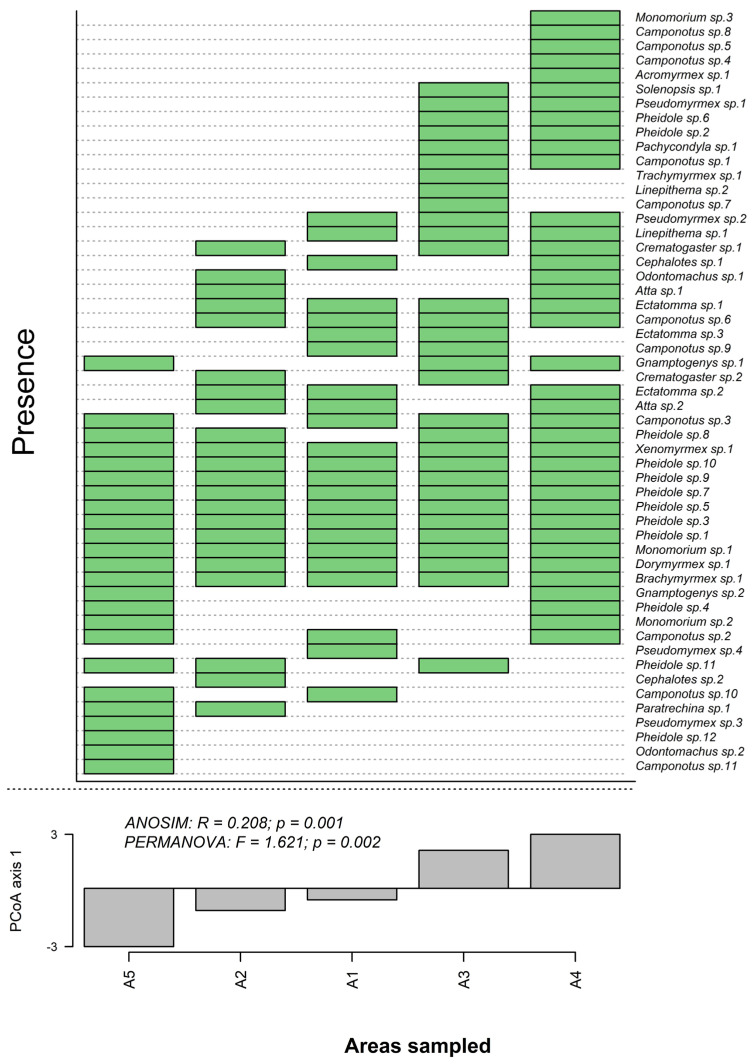
Dissimilarity in ant community composition (morphospecies) across the different forest fragments investigated was determined by Principal Coordinates Analysis (PCoA), Analysis of Similarities (ANOSIM), and Permutational Multivariate Analysis of Variance (PERMANOVA). A1: forest fragments located in Goiandira; A2, A3, A4, and A5: fragments in Ipameri.

For the pooled fragments, no significant difference was observed in the estimated richness of arboreal and edaphic ants (Jackknife 1, *p* > 0.05) (Figure 3a), and rarefaction curves (*p* > 0.05) corroborated this finding (Figure 3b). The wet season presented higher ant richness in the arboreal stratum. In contrast, in the ground stratum, no significant difference in ant richness was observed between the wet and dry climatic conditions (Jackknife, *p* > 0.05) (Figure 4). A dissimilarity in ant community composition (arboreal + epigeic) across the different investigated forest fragments was highlighted by PCoA, ANOSIM (R = 0.208; *p* = 0.001) and PERMANOVA (F = 1.621; R^2^ = 0.206; *p* = 0.001); Figure 5).

**Figure 6:**
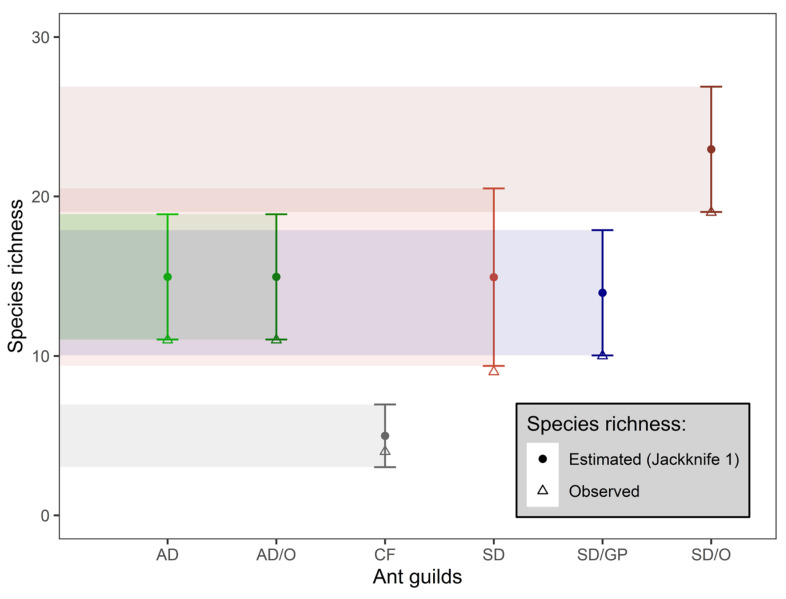
Ant richness for the five Cerrado fragments investigated in the southeastern region of Goiás. The evaluated guilds were as follows: CF = fungus-growing ants, AD = arboreal dominant ants, AD/O = dominant arboreal and omnivorous ants, SD = dominant ground ants, SD/O = dominant ground and omnivorous ants, SD/GP = dominant ground and generalist predatory ants. The bars in the figure represent the 95% confidence interval adopted by the Jackknife Estimator.

The SD/O guild (dominant ground ants/omnivorous ants) showed the highest estimated species richness, while the CF guild (fungus-growing ants) exhibited a significantly lower richness for this variable (Figure 6).

**Figure 7:**
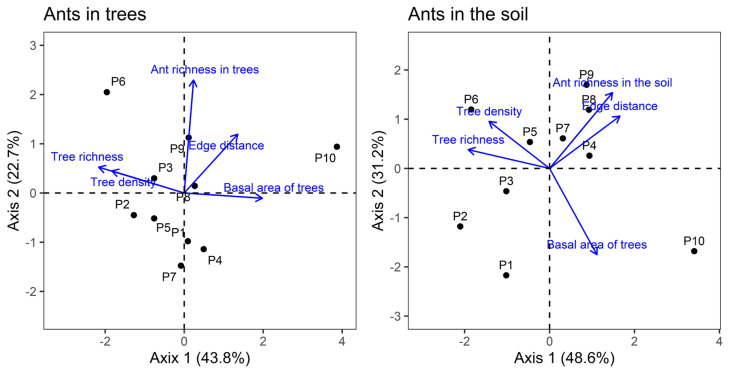
Principal Component Analysis (PCA) establishes the relationship between structuring variables of the Cerrado forest fragments and ant species richness. P1 to P10 represent the ant collection points along the transect, from the edge to the interior of the forest fragment, with traps spaced 10.0 m apart. Trap P1 (ground and tree) was placed at a distance of 5.0 m from the edge, and consequently, trap 10 (P10) was 95.0 m away from it.

Principal Component Analysis (PCA) revealed that points further into the fragment interior presented higher values of ant species richness and larger tree sizes (inferred from the tree trunk basal area) (Figure 7). The analysis used the average values for each sampling point. The sampling unit was the point; therefore, 10 samples and 5 variables were used. The *IndVal* analysis suggested two environmental quality indicator species for the ground stratum: *Pheidole* cf. *radoszkowskii* (Mayr, 1884) (*IndVal* = 0.595; *p* = 0.025) and *Crematogaster stollii* (Forel, 1885) (*IndVal* = 0.449; *p* = 0.01). Initially, both species were identified only by genus in morphotype, but they were determined to be species-specific and therefore considered environmental indicator species.

For the soybean landscape, the group with more than 50% soybean (A4 and A5) showed a relationship with *Camponotus novogranadensis* (Mayr, 1870) (*IndVal* = 0.738; *p* = 0.025), while the group with less than 50% soybean (A1, A2 and A3) showed a relationship with *Ectatomma tuberculatum* (Olivier, 1792) (*IndVal* = 0.660; *p* = 0.020) and *Crematogaster stollii* (Forel, 1885) (*IndVal* = 0.627; *p* = 0.025). For the forest landscape, the group with an area with more than 29% forest (A2) showed a relationship with *Crematogaster* sp1 (*IndVal* = 0.770; *p* = 0.010) and *Crematogaster stollii* (Forel, 1885) (*IndVal* = 0.903; *p* = 0.005). For the group of areas with less than 29% forest (A1, A3, A4, and A5), a relationship was found only for *Camponotus novogranadensis* (Mayr, 1870) (*IndVal* = 0.764; *p* = 0.05).

For savanna, in areas with more than 8% savanna, there was a relationship with *Crematogaster* sp1 (*IndVal* = 0.679; *p* = 0.015), *Crematogaster stollii* (Forel, 1885) (*IndVal* = 0.627; *p* = 0.025), *Atta sexdens* (Linnaeus, 1758) (*IndVal* = 0.577; *p* = 0.015), and *Ectatomma tuberculatum* (Olivier, 1792) (*IndVal* = 0.552; *p* = 0.025). For the pasture landscape, no species were found to be significantly related.

**Figure 8:**
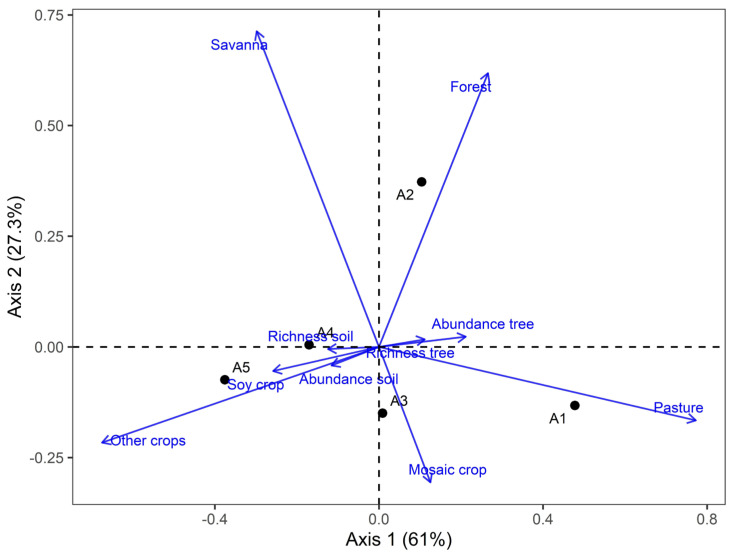
Correspondence Analysis (CA) establishes the relationship between landscape land use variables and the richness and abundance (capture frequency) attributes of arboreal and ground ants. A1 to A5 represent the sampling units from each collected location.

Once the structural patterns of the vegetation in each fragment were understood, we also sought to identify relationships between the landscape land use and the ant community. The Chi-square test of independence indicated a significant association between land use variables and ecological aspects of the ants (χ^2^(df = 36) = 160.57; *p* = 0.001). These results justify the application of Correspondence Analysis (CA) to investigate the structure of the association among categories. The findings demonstrate the influences of pasture land use, crop mosaics, other crops, and savanna and forest formations. Most of the collection areas are associated with agricultural land uses, with only A2 showing a greater relationship with forest areas (Figure 8). Among the ecological aspects of the ants, arboreal ant abundance and richness were related to the percentage of pasture in the areas (especially A1), whereas ground ant richness and abundance were related to the percentage of area with soybean and other crops (A4 and A5).

## 4. Discussion

The entomofauna community responds to alterations in forest fragments and the landscape, driven by different land uses in the surrounding area. Characteristics such as the size and features of the vegetation fragments influence the ant community, even affecting ground and arboreal ant communities differently. This further reinforces the use of this group as bioindicators of environmental alteration in agroecosystem landscapes. It is widely recognized that habitat loss is among the most significant drivers of biodiversity decline worldwide; authors like Gibson et al. [50] warn that there is no substitute for primary tropical forests. Therefore, understanding the population dynamics of plants and animals in these forests or their remaining fragments is of fundamental importance. Our study organisms, ants, exhibit varied feeding and nesting strategies, are abundant in tropical ecosystems, and perform key ecological processes, which enhance their potential as biological indicators [14,22].

In our study, we characterized the arboreal and epigeic ant communities present in Cerrado fragments bordered by crops, related these communities to structural variables of the fragments during the dry and wet seasons, and suggested their use as environmental bioindicators. Studies conducted by Bernardes Júnior et al. [14] in Cerrado fragments (dry forest phytophysiognomy) in a region relatively close to our study site, using the same ant capture method, identified 52 ant species—practically the same as our study, which found 53 morphospecies. The most frequently captured genera were *Pheidole* (Myrmicinae) and *Camponotus* (Formicinae), which together accounted for 43.4% of the captured morphospecies. The hyperdiversity phenomenon of these two genera was described by Wilson [51].

The large confidence intervals established by the Jackknife 1 estimator indicated substantial variation in the estimated ant morphospecies richness across the tree and ground strata, among the forest fragments, and among the sampling points. This condition likely explains the lack of significant differences in ant richness among most fragments. It is worth adding that fragment A4, which presented the highest absolute ant richness, had a highly heterogeneous shape (a large edge area with less dense arboreal vegetation and a relatively smaller, denser interior region), unlike the other fragments, which were covered with denser and more uniform arboreal vegetation. Importantly, fragment A4 is the only Legal Reserve fragment located in a public area, visibly less affected by anthropogenic activity compared to the other fragments located on private properties (authors’ note). This fact suggests greater environmental heterogeneity and, consequently, higher ant richness, as heterogeneous environments are typically associated with higher species richness.

In continuous Cerrado regions (dry forest areas measuring around 6000 hectares) relatively close to our study area, Rodrigues et al. [25] reported a predominance of epigeic ants compared to the arboreal stratum. According to Campos et al. [52], this is due to the greater heterogeneity of the leaf litter and soil environment, which provides a greater diversity of food and shelter. In our study, however, no significant difference was found in the total ant richness between the arboreal and ground strata.

As a rule, climatic conditions influence ant diversity within a habitat [53,54]. In our study, climatic conditions significantly influenced the diversity of captured arboreal ants. Santiago et al. [26] highlighted that it is characteristic of the dry forest environment in the Goiás Cerrado region for trees to partially or completely lose their leaves during the dry season. This could reduce the available food resources and even discourage bait exploration by foraging ants to avoid potential predators, thus resulting in lower species richness collected during the dry period. However, the capture frequency of epigeic ants was not influenced by the evaluated climatic conditions.

The Principal Coordinates Analysis (PCoA) revealed significant dissimilarity in the ant community among the fragments (see Figure 6). However, greater arboreal and epigeic ant richness in the innermost region of the fragment is shown in the PCA (see Figure 7). Generally, edge distance influences the composition of ant communities [4,14,55,56].

The term “guild,” according to Terborgh and Robinson [57], refers to a group of species that derive their subsistence from the same types of resources and use similar occupational strategies. Guild models, similar in part to those presented in our study, have been suggested as a different way to represent the structure of local ant communities in Brazilian Cerrado and Atlantic Forest environments [58]. Our study presented ant richness (genera) within their probable guilds, following the suggestions of Andersen et al. [38], Delabie et al. [39], Silvestre et al. [40], and Bernardes Júnior et al. [14].

The dominant guild, which stood out significantly from the others, was the dominant ground/omnivorous (SD/O) guild, while the least frequently captured was the fungus-growing (CF) guild. According to Delabie and Fowler [59], 50% of the myrmecofauna is associated with leaf litter on the soil surface in tropical forests. Therefore, higher ant richness is generally found in this stratum. Corroborating our data, Rodrigues et al. [25] also observed higher ant richness on the ground than in trees in the Goiás Cerrado, even when considering large trees.

It is important to highlight that the SD/O guild includes *Pheidole* species, which were represented by the highest number of morphospecies and were the most frequently captured in traps in both the arboreal and ground strata. However, the specificity of the bait method must be explicitly considered when interpreting richness estimates and *IndVal* results. Because baits primarily attract protein and carbohydrate-foraging ants, they systematically under sample guilds that do not regularly visit such resources. This directly affects the Jackknife 1 richness estimator, which relies on species incidence and rarity patterns: the low capture frequency of the CF guild leads to an underestimation of its true richness and biases the species accumulation curves. Similarly, *IndVal* results are influenced by bait specificity, potentially overestimating indicator values for bait-attracted guilds (e.g., SD/O) while failing to detect significant indicators among the CF guilds.

Furthermore, the CF guild—comprising *Atta* and *Acromyrmex*—was captured accidentally by the baited traps, as they forage primarily on fresh plant leaves rather than on bait offerings [52]. Thus, the adopted sampling method is not strictly suitable for them, and this incompatibility was explicitly confirmed and accounted for in our analyses: no inference on CF guild richness or indicator status is drawn beyond the observation of accidental capture. Consequently, comparisons with pitfall-trap studies must be qualified. Pitfall traps capture a broader spectrum of epigeic ant activity, including non-bait-attracted species, and generally yield higher representation of the CF guild. Our bait-based design therefore provides reliable comparisons among bait-sampled strata and periods but is not directly comparable to absolute richness estimates derived from pitfall-based protocols.

Despite the previous observation regarding the CF guild, we emphasize the relative efficiency of our rapid capture method. The total number of species captured (53) differs from the findings of Rodrigues et al. [25] and Bernardes Júnior et al. [14], who captured 66 and 60 species, respectively, in Cerrado areas close to our study site using pitfall traps or traps with the same bait used in our research. The difference found is probably due to the groups that are not frequently captured in the trap we used. Our study, while highly relevant in presenting some patterns of ant communities in the Cerrado, also has some methodological limitations that should be considered. The use of only one methodology and a limited sampling period is the main methodological limitation we present, since this can influence the profile of ants discovered. The approach with active bait mainly favors omnivorous and necrophagous species, such as the genus *Pheidole*, and reduces the frequency of fungus-cultivating ants (*Atta*, *Acromyrmex*), as previously mentioned.

The use of indicator species via the *IndVal* method is an important monitoring tool for conservation studies [60]. The *IndVal* analysis suggested two habitat indicator species for the ground stratum: *Pheidole* cf. *radoszkowskii* and *Crematogaster stollii*. Both species belong to the subfamily Myrmicinae, which is dominant in many tropical ecosystems [61]. For these two species in particular, their presence with significant capture frequencies, both in trees and in leaf litter, has already been recorded in the region surrounding our study area. The *Pheidole* genus has highly diverse feeding habits [62]. Workers of *P. radoszkoskii*, for instance, have been reported as predators of fly larvae on mammal carcasses, as well as scavengers collecting food from these carcasses for their colonies (necrophagous behavior) [63]. Therefore, using sardines as a bait attractant certainly contributed to their capture frequency data. The *Crematogaster* genus is described as a dominant arboreal group [40]. According to Longino et al. [60], *C. stollii* is a dominant arboreal species that typically nests in the trunks of living trees. Our study found workers of this ant foraging on both plants and leaf litter. However, their capture frequencies in trees did not present an adequate distribution pattern to be indicated as an environmental bioindicator for that stratum.

Regarding *IndVal* analyses considering landscape changes, the results obtained demonstrate that different ant species respond differently to variations in landscape cover, indicating that habitat structure acts as an ecological filter for the Formicidae assemblage. In the agricultural landscape, the predominance of soybeans (>50%) was associated with *Camponotus novogranadensis*, suggesting that this species can tolerate or even benefit from highly modified environments with low structural heterogeneity. In contrast, areas with less than 50% soybeans harbored *Ectatomma tuberculatum* and *Crematogaster stollii*, possibly due to the greater diversity of micro-habitats and resource availability in these locations. In the forest landscape, the relationship between *Crematogaster* sp1 and sp2 with areas of greater forest cover (>29%) reinforces the dependence of these species on more preserved conditions, such as higher humidity, leaf litter, and connectivity. On the other hand, *C. novogranadensis* was again associated with areas with lower forest cover (<29%), indicating that this species can be considered a generalist or even opportunistic, occupying more open or fragmented environments.

In the savanna landscape with areas with more than 8% cover, there was a greater richness of indicator species, including *Crematogaster* sp1 and *C. stollii*, *Atta sexdens*, and *E. tuberculatum*. This pattern suggests that even small remnants of savanna can maintain characteristic species, probably due to the presence of specific resources such as grass, exposed soil, and microclimate. The absence of significant indicator species for the pasture landscape may indicate that this environment is extremely homogeneous or degraded, limiting the occurrence of specialist species and favoring only generalist taxa already captured in other categories. Taken together, the data indicate that *Crematogaster* sp2 was the species with the highest indicator value in both preserved forests and savannas, suggesting ecological plasticity or a preference for more natural habitats. *C. novogranadensis* emerged as a strong indicator of anthropized environments (both soybean and degraded forests), potentially proving useful in environmental disturbance monitoring programs. The absence of indicators in pastures reinforces the need for more sustainable management in these areas for the conservation of ant fauna.

No species observed in our study were significantly selected as an environmental indicator for the arboreal stratum. Regarding the different land uses, the species found in this study are species that have a direct relationship with agricultural areas [25]. Therefore, considering the dynamics of this ant community, there is an indication for the use of these six species to be monitored in programs aimed at conserving or preserving these forest fragments. We emphasize the value of continuing to investigate the association found in this work, wherein ground ants are more strongly associated with agricultural transformations of the landscape, while arboreal ants are associated with forest and savanna formations. Soil arthropods may respond differently from arboreal arthropods, being uniquely influenced by the agricultural landscape [64]. Furthermore, it is relevant to understand the dynamics of social insects within agricultural landscapes and agroecosystems. Landscape management and alteration directly affect entomological communities, which can subsequently impact both the natural environment and agricultural production systems [65].

It is important to highlight that while various structural attributes of a fragment can be relatively easy to measure, they may not necessarily capture the dynamic properties of an ecosystem that represent its current performance [66]. Regarding land use and agroecosystem landscape alterations, previous studies have demonstrated that an increase in agricultural landscapes and a reduction in natural landscapes (forests) decrease the community of beneficial insects and increase the number of insects better adapted to agricultural conditions, as seen in the entomological community of vegetable gardens in Chile [20]. Our study, along with others conducted at a local scale, can serve as a reference for regional-scale research to better understand the anthropogenic impacts of Cerrado fragmentation. It remains crucial, whenever possible, to increase the landscape scale of studies, incorporating new monitoring activities with greater landscape diversity and over longer temporal scales.

## Figures and Tables

**Table 1:** Ants captured in five fragments of the Dry Forest, located in the municipalities of Goiandira and Ipameri, Southeast region of Goiás, Brazil.

Taxa	Guild *	Number of Morphospecies	Capture Frequency (%)			
			Rainy Season		Dry Season	
			Soil	Tree	Soil	Tree
*Acromyrmex*	CF	01	0.66	0	0	0
*Atta*	CF	02	0	0	4	2
*Brachymyrmex*	SD/O	01	6	4	4	3.33
*Camponotus*	AD/O	11	8.6	6.66	15.3	6
*Cephalotes*	AD	02	0	0.66	0	0.66
*Crematogaster*	AD	02	2.66	4.66	3.33	1.33
*Dorimyrmex*	SD	01	4.66	2.66	6	2
*Ectatomma*	SD/GP	03	8	4	0.66	0
*Gnamptogenys*	GP	02	0	1.33	0.66	0.66
*Linepthema*	SD	02	0	1.33	2	2.08
*Monomorium*	AD	03	6.66	2	2.66	1.33
*Odontomachus*	SD/O	02	1.33	0	0.66	0
*Pachycondyla*	SD/GP	01	0	0.66	0	0
*Paratrechina*	SD	01	1.33	0	0.66	0
*Pheidole*	SD/O	12	72	29.3	44.6	9.33
*Pseudomyrmex*	AD	04	0	0.66	2	2
*Solenopsis*	SD/O	01	0	0	3.33	0
*Trachymyrmex*	CF	01	0.66	0	0.66	0
*Xenomyrmex*	SD	01	10.6	3.33	2.66	2.66
**Total morphospecies**		53				

* Specialization or guild, modified from Andersen et al. [38], Delabie et al. [39], and Silvestre et al. [40]. SD = Soil dominant; GP = Generalist predators; AD = Arboreal dominant; O = Omnivores; CF = Cultivators of fungi.

## Data Availability

The original contributions presented in the study are all included in the article/Supplementary Materials. Further inquiries can be directed to the corresponding author.

## Supplementary Materials

The following supporting information can be downloaded at: https://www.mdpi.com/article/10.3390/insects17060656/s1, Table S1: List of tree species located by forest fragment. Table S2: Percentages of different land cover classes, provided by Collection 9 of the MapBiomas Project, which were calculated considering a radius of 1 km around the sampling locations. Fragment A1 is in the Municipality of Goiandira, GO, and fragments A2, A3, A4, and A5 are in the Municipality of Ipameri, GO.
