# Supplementary material for: Structure of Epigeic and Arboreal Ant Communities in Forest Fragments Within Agricultural Landscapes of the Brazilian Cerrado
Source: Insects. 2026 Jun 22;17(6):656. doi: 10.3390/insects17060656 (PMC13299821; doi:10.3390/insects17060656)
Supplement: Supplementary file 1 [file insects-17-00656-s001.zip › insects-4333439-supplementary.pdf]

insects

MDPI

Supplementary Materials:

Table S1: List of tree species located by forest fragment.

Tree species per dry forest fragment				
**A1 (Goiandira, GO)**	**A2 (Ipameri, GO)**	**A3 (Ipameri, GO)**	**A4 (Ipameri, GO)**	**A5 (Ipameri, GO)**
Myrcia rostrata	*Machaerium opacum*	*Rhamnidium elaeocarpum*	*Byrsonima pachyphylla*	*Terminalia glabrescens*
Maytenus floribunda	*Xilopia aromatica*	*Myracrodruon urundeuva*	*Cecropia pachystachya*	*Myracrodruon urundeuva*
Erythroxylum daphnites	*Qualea parviflora*	*Diospyros hispida*	*Annona coriacea*	*Inga edulis*
Schefflera macrocarpa	*Qualea grandiflora*	*Coccoloba mollis*	*Xilopia aromatica*	*Piptadenia gonoacantha*
Qualea grandiflora	*Pera glabrata*	*Maprounea guianensis*	*Roupala montana*	*Tapirira obtusa*
Rapanea ferruginea	*Matayba guianensis*	*Sclerolobium paniculatum*	*Sclerolobium paniculatum*	*Chrysophyllum emarginatum*
Pera glabrata	*Siparuna guianensis*	*Anadenanthera macrocarpa*	*Qualea grandiflora*	*Licania apetala*
Plathymenia reticulata	*Sclerolobium paniculatum*	*Aspidosperma polyneuron*	*Qualea parviflora*	*Cardiopetalum calophyllum*
Siparuna guianensi	*Magonia pubescens*	*Dimorphandra mollis*	*Diospyros hispida*	*Siparuna guianensis*
Machaerium acutifolium	*Myracrodruon urundeuva*	*Bauhinia forficata*	*Myracrodruon urundeuva*	*Licania kunthiana*
Eriotheca pentaphylla	*Terminalia glabrescens*	*Myrsine gardneriana*	*Caryocar brasiliense*	*Xylopia aromatica*
Acosmium dasycarpum	*Myrsine gardneriana*	*Myrsine coriacea*	*Maprounea guianensis*	*Virola sebifera*
Rudgea viburnoides	*Diospyros brasiliensis*	*Guazuma ulmifolia*	*Plathymenia reticulata*	*Coussarea hydrangaefolia*
Dipteryx alata	*Anadenanthera peregrina*	*Chrysophyllum marginatum*	*Kielmeyera coriacea*	*Bauhinia rufa*
Annona crassiflora	*Alibertia edulis*	*Hyeronima alchorneoides*	*Terminalia argentea*	*Anadenanthera macrocarpa*
Luehea divaricata	*Terminalia argentea*	*Cordiera sessilis*	*Connarus suberosus*	*Aspidosperma discolor*
Pterodon emarginatus	*Tabebuia roseoalba*	*Xylopia aromatica*	*Aegiphilla verticilata*	*Terminalia argentea*
Zanthoxylum rhoifolium	*Lithraea molleoides*	*Tapirira guianensis*	*Leptolobium dasycarpum*	*Eriotheca pentaphylla*
Matayba guianensis	*Aspidosperma discolor*	*Siparuna guianensis*	*Aspidosperma tomentosum*	*Matayba elaeagnoides*
Roupala montana	*Cordiera sessilis*	*Guettarda viburnoides*	*Lafoensia pacari*	*Hirtella gardneri*
Solanum sanctaecatharinae	*Dimorphandra mollis*	*Matayba elaeagnoides*	*Virola sebifera*	*Myrcia tomentosa*
Anadenanthera colubrina	*Coccoloba mollis*	*Ocotea corymbosa*	*Tapirira guianensis*	*Emmotum nitens*
Sclerolobium paniculatum	*Guettarda viburnoides*	*Leandra dasytricha*	*Chrysophyllum gonocarpum*	*Tapirira guianensis*
Sclerolobium aureum	*Maytenus floribunda*	*Ficus elastica*	*Dalbergia miscolobium*	*Guazuma ulmifolia*
Tabebuia aurea	*Emmotum nitens*	*Pera glabrata*	*Myrcia splendens*	*Cupania vernalis*
Guazuma ulmifolia	*Aspidosperma cuspa*	*Magnolia ovata*	*Machaerium acutifolium*	*Anadenanthera peregrina*
Pseudobombax tomentosum	*Diospyros hispida*	*Tapirira obtusa*	*Matayba elaeagnoides*	*Dilodendron bipinnatum*
Solanum lycocarpum	*Kielmeyera coriacea*	*Piper arboreum*	*Vatairea macrocarpa*	*Ocotea aciphylla*
Celtis iguanea	*Luehea grandiflora*	*Dicksonia sellowiana*	*Hirtella gracilipes*	*Cariniana estrellensis*
Tapirira guianensis	*Curatella americana*	*Xylopia emarginata*	*Copaifera langsdorffii*	*Hirtella glandulosa*
Agonandra brasiliensis	*Platypodium elegans*	*Calophyllum brasiliense*	*Emmotum nitens*	*Guatteria australis*
Psidium guajava	*Astronium fraxinifolium*	*Euterpe edulis*	*Coccoloba mollis*	*Eriotheca candolleana*
Casearia rupestris	*Tapirira guianensis*	*Protium heptaphyllum*	*Hirtella glandulosa*	*Tapura amazonica*
Crysophyllum marginatum	*Sclerolobium aureum*	*Licania apetala*	*Erythroxylum daphnites*	*Pouteria speciosa*
Cordiera sessilis	*Agonandra brasiliensis*	*Hymenaea stigonocarpa*	*Qualea multiflora*	*Alibertia sessilis*
Apuleia leiocarpa	*Aspidosperma subincanum*	*Magonia pubescens*	*Siparuna guianensis*	*Styrax camporum*

Myrcia splendens	*Cupania vernalis*		*Vochysia rufa*	*Maytenus floribunda*
Luehea grandiflora	*Ixora brevifolia*	*Qualea grandiflora*	*Diospyros burchellii*	*Cordiera macrophylla*
Hirtella gracilipes	*Pseudobombax tomentosum*	*Miconia*	*Annona crassiflora*	*Nectandra membranacea*
Hirtella glandulosa	*Rhamnidium elaeocarpum*	*Pterodon pubescens*	*Bowdichia virgilioides*	*Diospyros hispida*
Rhaminidium elaeocarpum	*Chrysophyllum gonocarpum*	*Virola urbaniana*	*Piptocarpha rotundifolia*	*Aspidosperma subincanum*
Tapirira obtusa	*Myrcia splendens*	*Symplocos nitens*	*Byrsonima spectabilis*	*Matayba guianensis*
Aspidosperma camporum	*Chrysophyllum splendens*	*Myrcia splendens*	*Machaerium brasiliensis*	*Amaioua edulis*
Coccoloba mollis	*Virola sebifera*	*Curatella americana*	*Alibertia edulis*	*Luehea grandiflora*
Dilodendrun dipinata	*Roupala montana*	*Erythroxylum daphnites*	*Cordiera sessilis*	*Copaifera langsdorffii*
Myrsine umbellata	*Inga cylindrica*	*Terminalia glabrescens*	*Byrsonima laxiflora*	*Ouratea castaneifolia*
Diospyros hispida	*Dilodendron bipinnatum*	*Tabebuia roseoalba*	*Casearia rupestris*	*Piptadenia rigida*
Sp.1 (Not identified)	*Cheiloclinium cognatum*	*Aspidosperma discolor*	*Lithraea molleoides*	*Cheiloclinium cognatum*
	Cecropia pachystachya	*Coussarea hydrangeifolia*	*Anadenanthera colubrina*	*Unonopsis guatterioides*
	Hirtella glandulosa	*Styrax oblongus*	*Cordiera macrophylla*	*Hymenaea courbaril*
	Licania apetala	*Emmotum nitens*	*Anadenanthera peregrina*	*Pouteria torta*
	Schefflera morototoni	*Machaerium villosum*	*Dimorphandra mollis*	*Roupala montana*
	Lamanonia ternata	*Psidium rufum*	*Astronium fraxinifolium*	*Metrodorea stipularis*
	Ocotea corymbosa	*Pouteria rivicoa*	*Platypodium elegans*	*Myrcia multiflora*
	Guazuma ulmifolia	*Ormosia arborea*	*Chrysophyllum marginatum*	*Ocotea spanantha*
	Tapirira obtusa	*Lauraceae sp.*	*Symplocos nitens*	*Protium spruceanum*
	Rudgea viburnoides	*Guatteria australis*	*Sclerolobium aureum*	*Ocotea spixiana*
	Styrax ferrugineus	*Aspidosperma ramiflorum*	*Rudgea viburnoides*	*Anadenanthera colubrina*
	Hymenaea stigonocarpa	*Unonopsis guatterioides*	*Callisthene major*	*Bocageopsis mattogrossensis*
	Bowdichia virgilioides	*Vatairea macrocarpa*	*Matayba guianensis*	*Aspidosperma parvifolium*
	Cecropia pachystachya	*Virola sebifera*	*Diospyros brasiliensis*	*Maprounea guianensis*
	Annona crassiflora	*Pterodon emarginatus*	Sp.4 (Not identified)	*Nectandra lanceolata*
	Symplocos nitens	*Myrcia splendens*		*Cecropia hololeuca*
	Eugenia myrcianthes	Sp.3 (Not identified)		*Pterodon pubescens*
	Erythroxylum daphnites			*Myrtaceae sp.*
	Eugenia dysenterica			*Roupala brasiliensis*
	Diospyros burchellii			*Pseudobombax endecaphyllum*
	Byrsonima pachyphylla			*Apuleia leiocarpa*
	Cordiera sessilis			*Senna macranthera*
	Sp.2 (Not identified)			*Magnolia ovata*
				Ocotea velutina
				Ixora breviflora
				Aspidosperma cylindrocarpon
				Ormosia arborea
				Trichilia silvatica

Table S2: Percentages of different land cover classes, provided by Collection 9 of the MapBiomas Project, which were calculated considering a radius of 1 km around the sampling locations. Fragment A1 is in the Municipality of Goiandira, GO, and fragments A2, A3, A4, and A5 are in the Municipality of Ipameri, GO.

Fragments/Areas	Fragment size (Ha)	Forest (%)	Savannah (%)	Pasture (%)	Mosaic of Uses (%)	Soybean (%)	Other crops (%)
A1	8.3	12.72	0	44.28	9.71	32.79	0.05
A2	53.7	29.39	8.32	16.71	3.77	41.78	0
A3	258.5	7.85	1.10	20.12	12.75	57.81	0.16
A4	42.5	8.06	8.06	8.69	6.74	66.37	0.85
A5	21.3	7.90	3.23	3.98	6.93	76.97	0.44
